# Leprosy Diagnostic Test Development As a Prerequisite Towards Elimination: Requirements from the User’s Perspective

**DOI:** 10.1371/journal.pntd.0004331

**Published:** 2016-02-11

**Authors:** Edith Roset Bahmanyar, William Cairns Smith, Patrick Brennan, Ray Cummings, Malcolm Duthie, Jan Hendrik Richardus, Paul Saunderson, Tin Shwe, Steven Rosen, Annemieke Geluk

**Affiliations:** 1 Novartis Foundation, Novartis Campus, Basel, Switzerland; 2 School of Medicine, University of Aberdeen, Aberdeen, Scotland, United Kingdom; 3 Department of Microbiology, Immunology and Pathology, Colorado State University, Fort Collins, Colorado, United States of America; 4 PATH, Seattle, Washington, United States of America; 5 Infectious Disease Research Institute, Seattle, Washington, United States of America; 6 Department of Public Health, Erasmus MC, University Medical Center Rotterdam, Rotterdam, The Netherlands; 7 American Leprosy Missions, Greenville, South Carolina, United States of America; 8 American Leprosy Missions, Yangon, Myanmar; 9 Novartis Pharmaceuticals Corporation, East Hanover, New Jersey, United States of America; 10 Department of Infectious Diseases, Leiden University Medical Center, Leiden, The Netherlands; Fondation Raoul Follereau, FRANCE

## Introduction

Leprosy is the complex disease manifestation of *Mycobacterium leprae* infection. Although prevalence has declined from 5.2 million globally in the 1980s, new annual case detection rates (CDRs) remain high, at more than 200,000 new cases per year [1], indicating that additional leprosy control strategies are required to halt transmission.

An Expert Meeting held in June 2013 in Geneva discussed strategies to transition from control to elimination and concluded that any viable programme would need to include: (i) early diagnosis and prompt multidrug therapy (MDT) for all patients, (ii) tracing and postexposure prophylaxis (PEP) for contacts of newly diagnosed patients, and (iii) strict epidemiological surveillance and systems to monitor progress [2]. Improved diagnostic tools would be of great value to achieve these goals.

A subsequent international Expert Panel met, with the goal to define the required attributes of a diagnostic test for leprosy that would support and facilitate leprosy elimination efforts in terms of complete interruption of transmission of *M*. *leprae*. A tool for identifying leprosy cases (asymptomatic and any symptomatic form of leprosy) was identified as a prerequisite to elimination, thereby addressing the goals of the 2020 London Declaration on Neglected Tropical Diseases [3,4]. However, given the challenges of developing such a diagnostic test, a two-step strategy, starting with a confirmatory test for clinical diagnosis among symptomatic patients, was considered as a pragmatic approach. This article presents the considerations, target population, target profile, and current research activities for leprosy diagnostic tools from a user’s perspective.

## Considerations for Development and Distribution of a Leprosy Field Test

Currently, leprosy is mainly diagnosed by expert clinicians using defined criteria, along with the use of slit-skin smears and biopsies [5]. As the prevalence of the disease is decreasing, clinical expertise is diminishing, leading to extended delays between onset of clinical signs and diagnosis and consequent maintenance of transmission of *M*. *leprae*. Hence, efforts to achieve elimination are undermined. In recognition of the need to move from leprosy control to preventing infection, an ideal test would identify *M*. *leprae*-infected individuals at risk of developing disease and/or who contribute to transmission. However, given the challenge of developing such a test in the absence of a gold standard, a two-step approach could prove to be a more expeditious strategy: first, obtaining a test to help health care workers in their clinical diagnosis and decision-making process for treatment while, over a longer term, another test to identify infected individuals would be developed. As part of the requirements, two intended uses (IU) for the tests were defined, based on end-user requirements (Table 1).

**Table 1:** Intended Use statements for a leprosy diagnostic test.

INTENDED USE I (IU1):	A leprosy diagnostic test designed to act as an aid in the diagnosis of symptomatic patients for whom the health care worker requires an independent diagnostic assessment
INTENDED USE 2 (IU2): Ideal test	An *M*. *leprae* test designed to diagnose individuals who have been infected with *M*. *leprae* and are destined to have clinical symptoms (asymptomatic person at risk of developing leprosy and sustaining transmission of *M*. *leprae*)

Additionally, diagnostic tests should respond to identified needs and consider: (1) endorsement by stakeholders as a requirement to progress to elimination, (2) adding value to current leprosy programmes, (3) providing direct benefit to the users through accuracy and performance in the target population, and (4) being user-friendly to allow application at point of health care or community levels.

## Target Population for a Leprosy Diagnostic Test, Considering Assay Specificity and the Positive Predictive Value of the Results

Receiving a leprosy diagnosis bears significant social implications related to stigma and medical implications due to the long treatment duration. Therefore, deploying a diagnostic test (for IU1 or IU2) that is not perfect requires a coherent strategy to manage positive results. An example of recommendations for programme guidelines is defined in Table 2.

**Table 2:** Recommendations for the deployment of a diagnostic test that is not 100% specific.

• Target high-risk groups (for example, contacts of new cases)
• Define clear educational messages for those tested, including the meaning of test positivity
• Continue observation, clinical examination, and management for test-positive subjects
• Provide MDT for subjects who meet the diagnostic criteria based on clinical signs and symptoms
• Define optimal short-term treatment for test-positive, asymptomatic subjects

In low-prevalence settings, for any test not 100% predictive, it is likely that most positives will be false positives. Therefore, to improve the predictive value, high-risk groups, such as skin clinic attendees or household contacts, need to be identified and targeted for testing.

Table 3 estimates the positive predictive value (PPV) of true leprosy in high-risk settings, although the proportion of false positives would depend on the proportion of people actually infected.

**Table 3:** Example of positive predictive values (PPV) of a leprosy diagnostic test.

Sensitivity 75%	Reference clinic attendees			Household contacts		
Specificity 95%	(2% with leprosy)			(<1% with leprosy [5])		
	Leprosy	No. leprosy	PPV	Leprosy	No. leprosy	PPV
Diagnostic test results						
** Pos**	**150**	**490**	**23.4%** (95% CI 20.2, 26.9%)	**15**	**100**	**13.4%** (95% CI 7.5, 20.6%)
** Neg**	**50**	**9,310**		**5**	**1,900**	
**TOTAL**	**200**	**9,800**		**20**	**2,000**	

Alternatively, a two-step approach could be considered, whereby an initially high sensitivity test of at least 95% is followed by a highly specific test of ≥95%, targeting high-risk populations to minimise false positives.

## How Assay Sensitivity May Impact the Elimination Target

Based on SIMCOLEP (individual-based mathematical model), analyses targeting household contacts have shown that the effects on disease incidence in the whole population vary with type of intervention, such as contact tracing, provision of chemoprophylaxis, Bacillus Calmette-Guerin (BCG) vaccination, and early (preclinical) diagnosis [6]. The model has been developed to include the indirect effect of interventions targeting contacts on the transmission of *M*. *leprae* in the whole population [7].

Using the same model, different scenarios were explored for an IU2 test with a range of sensitivities (specificity does not impact transmission) as a tool for achieving elimination, using data based on a representative population [8]. Two different approaches to testing—total population and contact surveys—were analysed with different endemicity levels (new case detection rate [NCDR]: 25, 5, and 1 per 100,000), and additionally, one or two surveys (with a two-year interval) were modelled for the total population.

Comparing the impact of continuous contact testing with a one-time total population survey, along with the subsequent treatment of test-positive individuals, results suggested that NCDR would be more quickly reduced by a one-time population survey. Increasing sensitivity had an inverse relation to the NCDR. These preliminary results suggest that the optimal strategy is to consecutively test and treat, and a single-survey approach with a test sensitivity of at least 90% would be sufficient to reach elimination. After a single survey, elimination is reached after about ten years, and after two surveys, elimination is reached between five and ten years; in a two-survey approach, test sensitivities of 90%, 80%, and even 70% appear sufficient to reach elimination. Because of the numbers needed to be tested, and not taking into consideration the poor PPV for disease, a population survey approach is only favourable in a highly endemic situation.

## Programme Consideration and Different Diagnostic Test Devices Suitable for Leprosy-Endemic Areas

High-risk populations have been identified as an optimal target for a diagnostic test. This would require national leprosy programmes to intensify their surveillance systems in order to trigger prompt and targeted testing of high-risk clusters. Individual geographic information and spatial analysis have already been evaluated to define spatiotemporal patterns of leprosy [9], but they would need to become integrated into systematic national surveillance systems, requiring substantial investment. Introducing a new diagnostic test with IU2 could certainly help to achieve leprosy elimination, but it would require a strong commitment from policy makers and donors.

Point-of-care, noninvasive tests need to be considered for leprosy diagnostics—ideally, a rapid lateral flow qualitative (positive or negative) test using capillary blood or urine and including one or several test analytes. Other types of testing devices, depending on the selected diagnostic marker, could also be considered, such as the host nucleic acid amplification test (NAAT) [10], which proved to be a promising diagnostic tool for tuberculosis [11,12]. However, such tests would require further “engineering” research to develop testing platforms that provide an accurate quantitative readout, e.g., devices to test skin sensitivity, hydration measurement devices, or portable ultrasound devices to measure the size of nerves. Innovative methods of combining different tests may allow higher sensitivity and specificity to be achieved.

As the diagnostic test will be novel, manufactured, and distributed, the tool and any related instrumentation or software will require regulatory review in accordance with the country where it will be used. A successful launch will need political commitment for test recommendation and integration into health care systems, and appropriate training of end users will be vital, given the implications of a positive test result.

## Target Product Profile of a Leprosy Diagnostic Test

The required attributes for the two IUs of a leprosy diagnostic test, derived mainly from the results of the discussion in the meeting, are summarised in Table 4. The test would diagnose both Multi-bacillary (MB) and Pauci-bacillary (PB) forms of leprosy [5].

**Table 4:** Target product profile of a leprosy diagnostic test.

Requirement category	Optimal	Minimum
**Intended Use 1**		
Target population:	Patients with any skin lesion or peripheral neurologic defect	Leprosy suspects
Sensitivity %:	90	75
Specificity %:	95	80
Sample:	Whole blood capillary sample	Skin, image, whole blood, or serum sample
Results:	Qualitative	Quantitative
Device:	Lateral flow rapid diagnostic test (RDT)	Portable point of care device
**Intended Use 2: Ideal**		
Target population:	Total population in endemic areas (high-risk population)	At-risk individuals (i.e., contacts of leprosy patients)
Sensitivity %:	≥ 90	70
Specificity %:	> 95	90
Sample:	Whole blood capillary sample; urine	Whole blood, serum, or other body fluid
Results	Qualitative	Quantitative
Device:	Lateral flow (RDT)	Portable point of care device

## Biomarkers for Leprosy

A review of leprosy biomarkers reveals that the ideal diagnostic biomarker is not currently available to fulfill the requirements of the target product profile [13]. Past and ongoing research is covering markers for different ends of the leprosy spectrum. Lepromatous Leprosy (LL/BL) is characterised by a very robust antibody response, whereas Tuberculoid Leprosy (TT/BT) is characterised by hardly any humoral immunity but much stronger cellular immunity. In addition, *M*. *leprae*-infected individuals without disease symptoms may vary in their biomarker profile [14,15]. Both cellular and humoral immunity against *M*. *leprae* determine the outcome of infection. Thus, tests that simultaneously detect biomarkers specific for both types of immune responses are the targets for a test for detection of asymptomatic *M*. *leprae* infection and hence progression of infection to clinical disease [16].

Two previously characterised *M*. *leprae* antigens, leprosy IDRI diagnostic-1 (LID-1) and ND-O-BSA, appear to have utility and have been combined as a possible biomarker for LL/BL leprosy (NDO-LID) [17]. Serum antibody responses in leprosy patients correlated with the bacteriological index and Ridley–Jopling categorisation. LL/BL leprosy patients were distinguished with a high degree of sensitivity (95.7%) and specificity (93.2%). Additionally, the NDO-LID serological test has been shown to detect slightly larger proportions of BL/LL and TT/BT leprosy than the serology leprosy test detecting Immunoglobulin M (IgM) antibodies to *M*. *leprae*-specific phenolic glycolipid (PGL-I) (87.0% versus 81.7% and 32.3% versus 6.5%, respectively), and it also demonstrated improved specificity [18]. Use of these antigens in rapid test formats, coupled with a simple test reader platform, can provide consistent, objective, and quantifiable assessment, potentially facilitating wider use in nonspecialised settings. However, this assay does not or only weakly detects TT/BT individuals, similar to the anti-PGL-1 IgM antibodies, which is usually around 20%–40%.

In addition to humoral immunity, several *M*. *leprae* proteins and peptides have been identified as specific targets for cellular immunity against *M*. *leprae* [19, 20, reviewed in 21], some of which are currently used to measure the level of exposure to *M*. *leprae* [14] and thus the risk of infection and subsequent disease. Furthermore, extended investigations on cellular immune response [14] as well as genetic host biomarkers [9] are under investigation in current field trials [22], allowing future development of improved immunodiagnostic assays in terms of sensitivity and operational and sampling requirements [23,24].

Promising results have been obtained using different approaches such as serological metabolomics to unravel the biological pathways involved in the immunomodulation of leprosy [25]. Also, pathogen-based approaches have been explored, aimed at the development of assays for *M*. *leprae* detection [26]. Besides their use for diagnostic purposes, it is of note that new biomarker discovery approaches for leprosy also contribute to our understanding of its immune-pathologic mechanisms and will aid in the identification of therapeutic interventions.

## Conclusion

In conclusion, in the absence of a perfect test to detect all *M*.*leprae*-infected individuals, a diagnostic test to confirm leprosy disease at an early stage among symptomatic patients would be an acceptable and certainly useful shorter-term compromise. In parallel, it is critical that stakeholders continue promoting the concept that zero transmission is only attainable if *M*.*leprae* infection can be measured, and correspondingly invest in longitudinal research to identify biomarkers for the diagnosis of asymptomatic infection as well as for the risk of developing disease.
